# Epicardial adipose tissue in patients with and without COVID-19 infection

**DOI:** 10.1016/j.ahjo.2025.100548

**Published:** 2025-04-19

**Authors:** Alexander J. Küng, Iryna Dykun, Matthias Totzeck, Raluca Mincu, Lars Michel, Clemens Kill, Oliver Witzke, Jan Buer, Tienush Rassaf, Amir A. Mahabadi

**Affiliations:** a West German Heart and Vascular Center Essen, Department of Cardiology and Vascular Medicine, University Hospital Essen, Hufelandstr, 55, 45147 Essen, Germany; b Center for Emergency Medicine, University Hospital Essen, Essen, Germany; c Department of Infectious Diseases, West German Centre of Infectious Diseases, University Hospital Essen, Essen, Germany; d Institute of Medical Microbiology, University Hospital Essen, Germany

**Keywords:** Epicardial adipose tissue, COVID-19, Myocardial injury, Echocardiography

## Abstract

**Background:**

Acute COVID-19 infection frequently affects the cardiovascular system and causes acute myocardial injury. Epicardial Adipose Tissue (EAT), a visceral adipose tissue surrounding the myocardium and coronary arteries, has unique paracrine and endocrine effects, modulating the heart's inflammatory environment. Systemic inflammation stimulates TNF-α and Interleukin-6 secretion from EAT, contributing to cytokine storms and intensifying systemic responses. We aimed to determine whether EAT amount differs in patients with and without acute COVID-19 infection and myocardial injury.

**Methods:**

This study analyzed the CoV-COR registry cohort, conducted at the University Hospital Essen, including patients with symptoms suggestive of COVID-19 infection. The infection was confirmed by PCR. EAT thickness was measured by two-dimensional TTE.

**Results:**

A total of 296 patients (mean age 63.6 ± 17.26 years, 55.4 % male) were included. Patients with confirmed COVID-19 infection were younger, more frequently treated with antihypertensive medication, and had higher BMI and systolic blood pressures. Univariate logistic regression showed no association between EAT and myocardial injury 0.97 (0.74; 1.28, *p* = 0.82). A trend towards an association was observed between increasing EAT thickness and COVID-19 infection 1.25 (0.99; 1.59, *p* = 0.060). Adjusting for age and gender strengthened the association, with a 48 % (1.14; 1.93, *p* = 0.004) increased odds of COVID-19 infection per increase in EAT thickness. Multivariable regression yielded consistent effect sizes 1.47 (1.01; 2.16, *p* = 0.047).

**Conclusion:**

EAT thickness is associated with the presence of an acute COVID-19 infection but not with a myocardial injury. Further research is needed to assess if systemic viral infection induces dynamic changes in EAT.

## 1 Introduction

The coronavirus disease (COVID-19), caused by SARS-CoV-2, primarily affects the lungs, but leads to a high inflammatory burden in general. In most cases, COVID-19 only affects the lungs and induces mild symptoms, but in some cases, patients develop serious multiorgan manifestations in the cardiovascular-, gastrointestinal-, hematological- and neurological system [[1], [2], [3]]. Particularly male patients with cardiovascular diseases (CVD) or cardiovascular risk factors such as arterial hypertension, older age, obesity, especially visceral obesity, and diabetes have a higher risk for a severe or critical course of the infection. Cardiovascular diseases not only play a central role as a risk factor, but the cardiovascular (CV) system itself is often affected during a COVID-19 infection. The involvement of the CV system can manifest in myocardial injury, cardiac arrhythmia, heart failure, cardiomyopathy, thrombosis, coagulopathy, or cardiac arrest [4,5]. Myocardial injury (MI), determined as elevated troponin, was observed in 12 to 28 % of patients with COVID-19 infection and is associated with an increased mortality. CVD as comorbidity during an infection is associated with the highest case fatality rate of all preexisting comorbidity (10.5 %). If there is both myocardial injury and CVD, patients have the highest risk of a lethal outcome [1,[6], [7], [8]].

Obesity is a well-established risk factor for CVD and is associated with impaired survival in COVID-19 [9,10]. Epicardial adipose tissue (EAT) is a visceral adipose tissue surrounding the heart and the coronary vasculature [11]. Increased epicardial fat is associated with cardiac disease manifestation such as myocardial injury, acute coronary syndromes, myocarditis, arrhythmia, and heart failure [[12], [13], [14]]. Due to its paracrine effects and no fascia separating, EAT modulates inflammation in close proximity to the myocardium [15]. It secretes pro-inflammatory cytokines such as TNF-a and Interleukin-6 [16], which has been shown to worsen the course of a COVID-19 infection as “cytokine storm” [17,18]. It was hypothesized that visceral adipose tissues and especially EAT acts as a reservoir for viral spread and worsen the course of infection [18].

We suspect that EAT plays a central role in the course of a COVID-infection. In this pre-defined post-hoc analysis of the prospective CoV-COR registry, we aimed to determine, whether echocardiography derived quantification of EAT associates with myocardial injury in COVID-19 infection. In specific, we aimed to evaluate, whether the link between EAT and myocardial injury in COVID-19 positive patients differentiates from patients without COVID-19 infection.

## 2 Materials and methods

### 2.1 Study sample/patients

The present analysis is based on the cohort of the CoV-COR registry, which was designed to assess cardiovascular risk factors and cardiovascular diseases in hospitalized patients with suspected COVID-19 at the University Hospital Essen. We included all patients between April 2020 and March 2021 with symptoms suggestive of COVID-19 infection presenting to the Center of Emergency Medicine at the University Hospital Essen and confirmed the infection by PCR on a deep nasopharyngeal swab sample. Initially, all patients were treated by an interdisciplinary team of physicians in the emergency room. Admission criteria included hemodynamic instability, respiratory insufficiency, frailty, severe fatigue, and/or signs of organ failure. If hospital admission was considered necessary according to treating physician's discretion, COVID-positive patients were transferred to specialized COVID wards, whereas COVID-negative patients were transferred to respective specialty unit, based on the leading diagnosis. If the medical staff of the emergency department determined that there was no indication for inpatient admission, the patient was discharged to an outpatient setting.

All patients underwent a standardized transthoracic echocardiography (TTE) exam, an electrocardiogram, and standardized laboratory work- up including biobanking. Patients without TTE or troponin levels were excluded. In addition, patients were excluded if image quality was not sufficient for reliable quantification of EAT.

The study was carried out considering the ethical guidelines of the Helsinki Declaration and received the corresponding vote through the local ethic commission (20–9213-BO). All patients provided written informed consent.

### 2.2 Echocardiography and EAT quantification

**Fig. 1:**
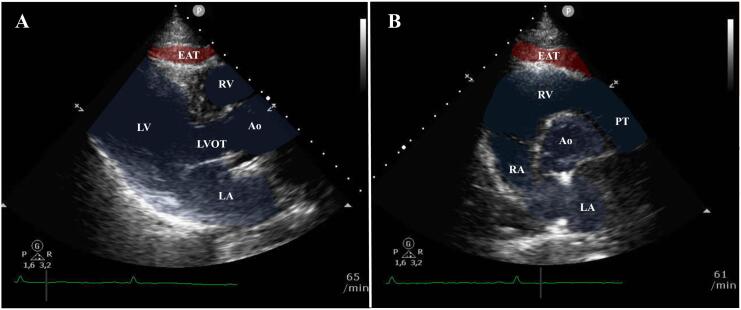
Epicardial Adipose tissue was quantified using transthoracic echocardiography. The region of interest was the end systolic space between the epicardial wall of the myocardium and the visceral layer of the pericardium in parasternal long (A) and short axis (B).

The EAT thickness was determined by two-dimensional TTE, which was taken and recorded during the hospitalization. If patients received more than one TTE, the first exam within the hospital stay was used. All EAT thickness measurements were performed offline by a single reader on a dedicated workstation using the Philips QLAB software Version 2.3 (Philips Healthcare, Amsterdam, the Netherlands). EAT was defined as the space between the epicardial wall of the myocardium and the visceral layer of the pericardium. Three measurements were taken, at least one of them in parasternal long axis and one in parasternal short axis. In each case the maximum in the end systole was counted as the value for the EAT (Fig. 1). From three measurements, the mean was determined as EAT thickness. Prior to this analysis, we evaluated the interobserver variability for EAT thickness in 20 cases and observed an excellent reproducibility (ICC 0.92, *p* < 0.0001) [19].

### 2.3 Risk factor and clinical assessment

Risk factors and clinical diagnoses were collected using standardized case report forms and questionnaires on admission. Clinical characteristics, laboratory values and all information regarding the intra-hospital course was retrieved from the hospital information system. Systolic and diastolic blood pressures were assessed from admission records. Hypertension was defined as systolic pressure > 140 mmHg or diastolic blood pressure of >90 mmHg or currently being on antihypertensive medication. The body mass index (BMI) was calculated as body weight divided by the square of height, as documented on admission. Total cholesterol, high-density lipoprotein (HDL)-, low- density lipoprotein (LDL)-cholesterol, l-lactatdehydrogenase (LDH), Troponin, N-terminal prohormone of brain natriuretic peptide (NT-proBNP), C-reactive protein (CRP), and procalcitonin (PCT) were evaluated using standardized enzymatic measures and recorded from the same hospital stay. Diabetes was defined based on fasting glucose levels, HbA1c levels, and medication. Active smoking, lung diseases, cancer, and a positive family history of premature coronary heart disease were assessed as documented by the treating physicians.

### 2.4 Endpoint definition

The primary endpoint is myocardial injury, defined as elevated cardiac troponin values (cTn) with at least one value above the 99th percentile upper reference limit (URL). A high-sensitive troponin I assay (Siemens Atellica, Erlangen, Germany) was used in all patients. Initial troponin was determined as part of the study protocol, while serial troponin evaluation was applied according to the discretion of treating physicians [20].

### 2.5 Statistical analysis

Continuous variables were presented as mean ± standard deviation and as median (interquartile range) if non-normally distributed and binary variables as frequency (%). Baseline characteristics were stratified by subgroups with and without confirmed COVID-19 infection as well as in subgroups with and without myocardial injury. Differences were compared using a two-sided *t*-test or Mann-Whitney *U* test for continuous variables and Fisher's Exact test for categorical variables. The thickness of the EAT was normally distributed. Logistic regression analysis was used to determine the association of myocardial injury and EAT thickness and in a second analysis of an acute COVID-19 infection and the EAT thickness. Further, logistic regression analysis was used to evaluate the association of EAT and myocardial injury in the cohort of COVID-19 positive patients.

Risk factor adjustment was performed as follows: (i) unadjusted, (ii) adjusted for age and sex, and (iii) age, sex, smoker, diabetes, BMI, hypertension, and dyslipidemia. For the regression analyses, effect sizes were determined for each standard deviation increase of EAT thickness.

Lastly, mortality rates were compared between patients with and without acute COVID-19 infection in an exploratory analysis using the Chi-Square test. For the subgroup analysis, Fisher's Exact test was applied to compare mortality rates between patients with and without myocardial injury, stratified by infection status.

All analyses were performed using IBM SPSS Statistics Version 28.0.0.0 (IBM Corporation, Armonk, United States of America), A *p* value of <0.05 indicated statistical significance.

## 3 Results

Overall, a total of 296 patients suggestive of COVID-19 Infection (164 males, 132 females; mean age 63.6 ± 17.26 years) were included in the analysis. Detailed patient baseline characteristics are depicted in Table 1. Patients with confirmed COVID-19 Infection were younger, had higher BMI and higher systolic blood pressures, and were more often treated with antihypertensive medication, whereas the total cholesterol, the rate of lipid lowering medication, the total NT-pro-BNP, procalcitonin (PCT) and white blood cell (WBC) count was higher in the group of patients without COVID-19. Patients with elevated Troponin were more often treated with antihypertensive and lipid lowering medication, had a higher NT-pro-BNP levels, and higher WB count.

**Table 1:** Baseline characteristics for the entire cohort and stratified by elevated Troponin or normal Troponin and COVID-19 infection or non-COVID-19 infection.

	Overall (*n* = 296)	Elevated Troponin (*n* = 66)	Normal Troponin (*n* = 230)	*p*-value	Covid positive (*n* = 161)	Covid negative (*n* = 135)	p-value
Age (years) (*n* = 296)	63.6 ± 17.26	67.2 ± 16.39	62.5 ± 17.4	0.051	61.50 ± 17.03	66.07 ± 17.26	0.023
Male (%)	164 (55.4)	38 (57.6)	126 (54.8)	0.69	89 (55.3)	75 (55.6)	0.96
BMI (kg/m2) (*n* = 179)	27.1 ± 5.0	26. ± 4.57	27.4 ± 6.4	0.17	28.11 ± 7.1	26.1 ± 4.63	0.024
Syst. RR (mmHg) (*n* = 278)	127.1 ± 21.04	126.2 ± 20.86	127.4 ± 21.13	0.69	129.4 ± 21.73	124.2 ± 19.85	0.040
Diast. RR (mmHg)	72.3 ± 15.29	71.2 ± 14.02	72.6 ± 15.66	0.53	72.6 ± 14.03	71.8 ±16.8	0.66
Antihypertensive medication (%) (n = 296)	202 (68.2)	56 (84.9)	146 (63.5)	0.001	102 (63.4)	100 (74.1)	0.048
Beta-blocker	139 (47)	38 (57.6)	101 (43.9)	0.052	68 (42.2)	71 (52.6)	0.081
Diuretics	111 (37.5)	36 (54.5)	75 (32.6)	0.001	42 (26.1)	69 (51.1)	<0.001
ACE/ AT-1 Antagonists	98 (33.1)	31 (47)	67 (29.1)	0.008	41 (25.5)	57 (42.2)	0.003
Ca Chanel-blocker	78 (26.4)	24 (36.4)	54 (23.5)	0.04	36 (22.4)	42 (31.1)	0.112
Total cholesterol (mg/dl) (*n* = 100)	154.8 ± 51.88	148 ± 46.24	158 ± 54.37	0.37	135.4 ± 53.86	163.9 ± 48.69	0.009
LDL (mg/dl) (*n* = 84)	103.9 ± 45.07	96.44 ± 44.4	108.5 ± 45.29	0.24	116.3 ± 48.15	100.5 ± 43.97	0.19
HDL (mg/dl) (*n* = 83)	44.7 ± 25.59	41.4 ± 15.74	46.6 ± 29.74	0.38	46.4 ± 41.18	44.2 ± 19.15	0.75
Lipid-lowering medication (%) (*n* = 296)	106 (35.8)	37 (56.1)	69 (30)	<0.001	40 (24.8)	66 (48.9)	<0.001
Diabetes (%) (n = 296)	20 (6.8)	5 (7.6)	15 (6.5)	0.76	13 (8.1)	7 (5.2)	0.32

Prior cardiovascular disease (%) (n = 296)							
Coronary artery disease							
Prior PCI	42 (14.2)	21 (31.8)	21 (9.1)	<0.001	15 (9.3)	27 (20)	0.012
Prior ACB	19 (6.4)	9 (13.6)	10 (4.3)	0.018	6 (3.7)	13 (9.6)	0.055
Atrial Fibrillation	48 (16.2)	14 (21.2)	34 (14.8)	0.26	17 (10.6)	31 (23)	0.004
Heart Failure	58 (19.6)	28 (42.4)	30 (13)	<0.001	10 (6.2)	48 (35.6)	<0.001
Smoking (%) (n = 296)							
Current smoker	12 (4.1)	3 (4.6)	9 (3.9)	0.82	4 (2.5)	8 (5.9)	0.14
Former smoker	27 (9.1)	6 (9.1)	21 (9.1)	0.99	10 (6.2)	17 (12.6)	0.058
Never smoked	257 (86.8)	57 (86.4)	200 (87)	0.9	147 (91.3)	110 (81.5)	0.013
Pos. fam. History (%)							
NT-proBNP (pg/ml) (median [Q1, Q3]), (*n* = 211)	517 [149; 2505]	2795 [755; 7486]	282 [98.75; 1681.25]	<0.001	201 [96.5; 1038.5]	1169.5 [254.25; 5432.25]	<0.001
Inflammatory markers							
CRP (mg/l) (n = 277)	2.7 [0.9; 7.05]	2.95 [1.08; 9.55]	2.7 [0.8; 6.7]	0.3	3.3 [1.02; 7.1]	2.3 [0.55; 6.15]	0.13
PCT (ng/ml) (n = 228)	0.07 [0.03; 0.20]	0.11 [0.05; 0.38]	0.05 [0.03; 0.17]	<0.001	0.05 [0.03; 0.14]	0.11 [0.04; 0.39]	<0.001
WBC Count in thousand (n = 288)	7.31 ± 3.31	8.27 ± 3.18	7.03 ± 3.3	0.008	6.66 ± 3.12	8.04 ± 3.34	<0.001
Interleucine 6 (pg/ml) (n = 144)	23.00 [9.65; 61.68]	24.50 [12.40; 74.4]	22.40 [8.9; 54.55]	0.244	21.2 [8.57; 51.83]	32.05 [12.9; 71.05]	0.137

**Fig. 2:**
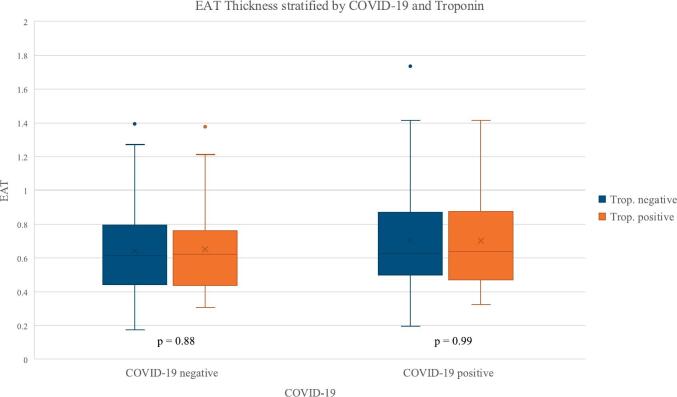
Epicardial Adipose tissue thickness stratified by COVID-19 infection status and Troponin levels. Patients are grouped into COVID-19 positive and negative cohorts, further subdivided into those with normal and elevated Troponin levels.

EAT thickness was not different in patients with vs. without myocardial injury nor comparing COVID-19 positive with COVID-19 negative patients (Table 2). Secondly, when patients were stratified by COVID-19 status, and the association between EAT thickness and myocardial injury was subsequently evaluated and no significant difference was observed (Fig. 2).

**Table 2:** EAT thickness for the entire cohort and the median age divided cohorts, stratified by elevated Troponin or normal Troponin and COVID-19 infection or non-COVID-19 infection.

	Overall (n = 296)	Elevated Troponin (n = 66)	Normal Troponin (n = 230)	p-value	COVID-19 positive (n = 161)	COVID-19 negative (n = 135)	p-value
EAT thickness (mm ± SD)							
All patients	0.676 ± 0.262	0.669 ± 0.253	0.678 ± 0.265	0.82	0.702 ± 0.271	0.644 ± 0.248	0.058
Age < 65.5 years		0.61 ± 0.236	0.61 ± 0.251	0.98	0.641 ± 0.259	0.542 ± 0.216	0.019
Age ≥ 65.5 years		0.71 ± 0.258	0.76 ± 0.258	0.26	0.788 ± 0.268	0.712 ± 0.246	0.08

To evaluate a crosslink between BMI, EAT thickness and presence of COVID-19 infection, we first evaluated the correlation between EAT and BMI. Supplementary Fig. 1 depicts the modest link between both measures. We stratified the overall cohort into an obese and non-obese group (BMI < 30 or ≥ 30). Within both groups, we again observed no difference in EAT thickness between COVID-19 positive or negative patients (BMI < 30: EAT thickness 0.672 ± 0.257 vs. 0.631 ± 0.221, *p* = 0.31and BMI ≥30: EAT thickness 0.745 ± 0.258 vs. 0.68 ± 0.273, *p* = 0.45, Supplementary Table 1).

As increasing age was linked with higher EAT thickness but mean age was lower in the group of patients with prevalent COVID-19 infection, we performed a subgroup analysis, stratifying be median age (<65.5 or ≥ 65.5 years). Here we observed that in the patients aged <65.5 years, patients with COVID-19 infection have more EAT as compared to patients without an acute infection. Patients aged ≥65.5 years had numerically higher EAT thickness when having an acute COVID-19 infection, which, however, did not reach statistical significance (Table 2).

**Fig. 3:**
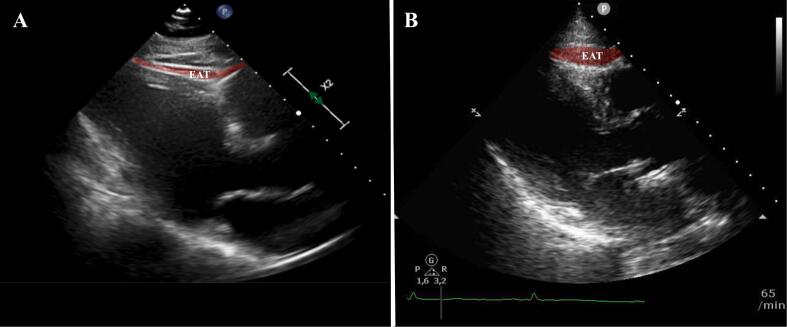
Epicardial Adipose tissue thickness in the parasternal long axis in a patient without acute COVID-19 infection (A) compared to a patient with acute COVID-19 infection (B).

In a univariate logistic regression analysis, there was a trend for the association of increasing EAT with acute COVID-19 infection (Table 3; Fig. 3). The association was strengthened when adjusting for age and gender with a 47 % increase in the odds of a COVID-19 infection per SD increase in EAT thickness. When ancillary adjusting for traditional cardiovascular risk factors, in a multivariable regression analysis, the effect remained stable.

**Table 3:** Univariate and multivariable logistic regression analysis for the prediction of COVID-19 infection.

Model	OR (95 % CI)	p value
EAT/ SD	1.25 (0.991–1.59)	0.060
+ Age + Sex	1.48 (1.14–1.93)	0.004
MV	1.47 (1.01–2.16)	0.047

Multivariable Analysis: Age, sex, EAT/SD, active smoker, diabetes, BMI, hypertension, dyslipidemia.

We evaluated the association between EAT thickness and presence of myocardial injury, but no trend emerged in unadjusted, age- and gender-adjusted and multivariable adjusted models (Table 4).

**Table 4:** Univariate and multivariable logistic regression analysis for the prediction of myocardial injury.

Model	OR (95 % CI)	*p* value
EAT/ SD	0.97 (0.74–1.28)	0.82
+Age + Sex	0.86 (0.63–1.16)	0.32
MV	0.98 (0.66–1.47)	0.93

Multivariable Analysis: Age, sex, EAT/SD, active smoker, diabetes, BMI, hypertension, dyslipidemia.

In the cohort of patients testing positive for COVID-19, the relationship between EAT thickness and the occurrence of myocardial injury was assessed; however, no pattern was observed in unadjusted, age- and gender-adjusted, or multivariable-adjusted analyses (Table 5).

**Table 5:** Univariate and multivariable logistic regression analysis for the prediction of myocardial injury in COVID-19 positive patients.

Model	OR (95 % CI)	p value
EAT/ SD	1.002 (0.66–1.51)	0.99
+Age + Sex	0.91 (0.58–1.42)	0.67
MV	0.81 (0.41–1.61)	0.55

Multivariable Analysis: Age, sex, EAT/SD, active smoker, diabetes, BMI, hypertension, dyslipidemia.

Follow-up data at 90 days were available for all 296 patients (100 %). Among these, 15 patients (5.07 %) died. The mortality rate did not differ significantly between patients with and without acute COVID-19 infection (4.55 % vs. 6.3 %; *p* = 0.54).

When stratified by the presence or absence of COVID-19 infection, comparable differences in mortality rates were observed between patients with and without myocardial injury. However, these differences did not reach statistical significance due to the low number of observations in each group (COVID-19 negative: 7.89 % vs. 5.62 %, *p* = 0.7; COVID-19 positive: 13.64 % vs. 3.03 %, *p* = 0.076).

## 4 Discussion

We examined the thickness of EAT as quantified by TTE in patients with and without COVID-19 infection and in patients with and without myocardial injury. The main findings are clearly summarized as follows: (i) patients with an acute COVID-19 infection have slightly more EAT than in patients without; (ii) adjustment for age and sex strengthened the association between EAT and COVID-19 infection, whereas effect sizes remained unchanged upon addition in multivariable regression analysis; (iii) EAT thickness was not linked with the probability of myocardial injury, neither in the overall cohort nor in the subgroup of COVID-19 positive patients. Our results suggest that an increase in EAT thickness is associated with an acute COVID-19 infection and its inflammatory state, whereas our findings showed no association between an increase in EAT thickness and myocardial injury.

Our findings align with previous studies examining COVID-19 and its impact on the CV system. The presence of CV risk factors or CVD were early identified as predictors of adverse outcomes in COVID-19 infection [21,22]. Obesity, defined as high BMI (BMI ≥ 30), is an established risk factor for CVD and as well for a severe course of an acute COVID-19 infection. Assessment of visceral adipose tissue may outperform quantification of BMI with respect to cardiovascular risk assessment [23,24]. Recent studies using CT imaging have shown a higher proportion of visceral fat being associated with an increased rate of severe courses and the need of intensive care and invasive mechanical ventilation during an acute COVID-19 infection [25]. These finding suggest that among cardiovascular risk factors, visceral obesity may support the inflammatory response in the setting of acute COVID-19 infection.

EAT as a visceral fat depot of the thorax considered of specific interest with respect to cardiovascular disease manifestation, as it surrounds the heart and the coronary arteries and has endocrine and paracrine activity, influencing the organs in a unique pathophysiological way [23]. EAT secretes several pro- and anti-inflammatory mediators and cytokines such as adiponectin, Il-6, and TNF α [16]. With increasing EAT thickness Adiponectin, which acts as a stabilizer of the inhibitor of, decreases in its quantity. Therefore, an enhanced activity of NF kappa B leads to an increase in TNF α [26,27]. This mismatch of pro- and anti-inflammatory markers leads to a pro-inflammatory milieu and is suspected to have a local influence on the underlying coronary segments and myocardium. Previous studies have already shown that an increase in EAT is associated with an increased rate of CV manifestations, such as HFpEF, coronary artery disease, atrial fibrillation, and aortic valve stenosis [19,28,29]. The causal mechanisms are likely multifactorial, combining direct endocrine effects of EAT and its known association with other cardiovascular risk factors.

Acute COVID-19 infection induces a systemic pro-inflammatory state characterized by a cytokine storm, which can lead to multiple organ manifestations. EAT, as an inflammation-amplifying fat depot surrounding the heart, may further intensify this cytokine storm by secreting pro-inflammatory mediators [30]. EAT may influence the course of an acute infection through its reservoir function for viral replication and worsen the course by augmenting the immune response [18]. In a retrospective meta-analysis the hypothesis of the crucial influence of increasing EAT and a severe course of an acute COVID-19 infection was supported. In 1128 patients during an acute COVID-19 infection higher EAT volume was associated with an increased rate of ICU admission and required more often an invasive mechanical ventilation [31]. Furthermore the link between EAT volume and myocardial injury revealed that patients with higher EAT volumes face a greater risk of myocardial damage and was also linked with an increased mortality rate [[32], [33], [34], [35]].

In contrast, our study did not find a significant correlation between increased EAT thickness and myocardial injury in the context of acute COVID-19 infection. However, our results support the hypothesis that acute COVID-19 infection locally affects visceral adipose tissue near the heart, thereby amplifying the cytokine storm. This vicious cycle may exacerbate the severity of the acute infection. Further research is required to investigate whether interventions targeting EAT reduction might mitigate the pro-inflammatory state observed during acute COVID-19 infections.

### 4.1 Limitations

Several caveats of the present analysis warrant further consideration. At first, the single center design only including patients presenting to a tertiary care center may have led to an underrepresentation of mild COVID-19 in the collective. Secondly, EAT was determined as the mean of diameter measurements from two-dimensional echocardiography. While these measures were highly reproducible, EAT thickness at the free right ventricular wall may not accurately determine the true three-dimensional EAT volume. Lastly, our results are based on a predominantly Caucasian cohort; hence generalization to other ethnic groups remains uncertain.

## 5 Conclusion

EAT thickness does not appear to be associated with acute myocardial injury in patients experiencing an acute COVID-19 infection. Nonetheless, an association was observed between increased EAT thickness and the presence of COVID-19 infection, suggesting a potential link with the inflammatory response characteristic of this condition. Further investigation is essential to clarify whether the heightened inflammatory state induced by COVID-19 directly impacts EAT thickness measured via TTE.

## Ethical-statement

The study was carried out considering the ethical guidelines of the Helsinki Declaration and received the corresponding vote through the local ethic commission (20–9213-BO). All patients provided written informed consent.

## Credit authorship contribution statement

**Alexander J. Küng:** Data curation, Methodology, Writing – original draft. **Iryna Dykun:** Data curation, Writing – review & editing. **Matthias Totzeck:** Data curation, Resources, Writing – review & editing. **Raluca Mincu:** Data curation, Writing – review & editing. **Lars Michel:** Data curation, Writing – review & editing. **Clemens Kill:** Data curation, Resources, Writing – review & editing. **Oliver Witzke:** Data curation, Resources, Writing – review & editing. **Jan Buer:** Data curation, Resources, Writing – review & editing. **Tienush Rassaf:** Investigation, Resources, Supervision, Writing – review & editing. **Amir A. Mahabadi:** Conceptualization, Formal analysis, Methodology, Project administration, Supervision, Writing – original draft.

## Declaration of competing interest

The authors declare no competing financial interests.
